# Increasing false positive diagnoses may lead to overestimation of stroke incidence, particularly in the young: a cross-sectional study

**DOI:** 10.1186/s12883-021-02172-1

**Published:** 2021-04-08

**Authors:** Abhinav J. Appukutty, Lesli E. Skolarus, Mellanie V. Springer, William J. Meurer, James F. Burke

**Affiliations:** 1 University of Michigan Medical School, 1500 E Medical Center Dr, Ann Arbor, MI 48109 USA; 2 Stroke Program, University of Michigan Medical School, Ann Arbor, MI 48109 USA; 3 School of Public Health, University of Michigan, 1415 Washington Heights, Ann Arbor, MI 48109 USA; 4 Emergency Department, University of Michigan Medical School, 1500 E Medical Center Dr, Ann Arbor, MI 48109 USA; 5 Department of Neurology, VA Ann Arbor Healthcare System, Ann Arbor, MI 48105 USA

**Keywords:** Stroke, Stroke in the young, Epidemiology, Diagnosis, False positive

## Abstract

**Background:**

Stroke incidence is reportedly increasing in younger populations, although the reasons for this are not clear. We explored possible reasons by quantifying trends in neurologically focused emergency department (ED) visits, classification of stroke vs. TIA, and imaging use.

**Methods:**

We performed a retrospective, serial, cross-sectional study using the National Hospital Ambulatory Medical Care Survey to examine time trends in age-stratified primary reasons for visit, stroke/TIA diagnoses, and MRI utilization from 1995 to 2000 and 2005–2015.

**Results:**

Five million eight hundred thousand ED visits with a primary diagnosis of stroke (CI 5.3 M–6.4 M) were represented in the data. The incidence of neurologically focused reason for visits (Neuro RFVs) increased over time in both the young and in older adults (young: + 111 Neuro RFVs/100,000 population/year, CI + 94 − + 130; older adults: + 70 Neuro RFVs/100,000 population/year, CI + 34 − + 108). The proportion of combined stroke and TIA diagnoses decreased over time amongst older adults with a Neuro RFV (OR 0.95 per year, *p* < 0.01, CI 0.94–0.96) but did not change in the young (OR 1.00 per year, *p* = 0.88, CI 0.95–1.04). Within the stroke/TIA population, no changes in the proportion of stroke or TIA were identified. MRI utilization rates amongst patients with a Neuro RFV increased for both age groups.

**Conclusions:**

We found, but did not anticipate, increased incidence of neurologically focused ED visits in both age groups. Given the lower pre-test probability of a stroke in younger adults, this suggests that false positive stroke diagnoses may be increasing and may be increasing more rapidly in the young than in older adults.

**Supplementary Information:**

The online version contains supplementary material available at 10.1186/s12883-021-02172-1.

## Background

Although the risk of stroke increases with aging, there are considerable data suggesting that stroke incidence is increasing in young adults. Studies report a national increase in hospitalizations for acute ischemic stroke from 2003 to 2012 in adults aged 35–54 across all races and ethnicities [1, 2]. Other reports with similar trends have been reported in the US and Europe [3–9].

The rising prevalence of cardiovascular risk factors in young adults is one possible explanation for the observed increase in stroke in the young [10]. The prevalence of obesity and other traditional vascular risk factors such as hypertension, diabetes, hyperlipidemia, and cigarette smoking have also been reported to be increasing in young adults [1, 11]. However, these trends may be in part due to differences in risk factor measurement over time [12], and it is unclear if the magnitude of these changes is large enough to account for the estimated increases in stroke incidence.

Thus, alternate theories for the observed trend in increasing stroke incidence in the young should be considered. One possible contributing factor is the increased use of advanced imaging. The increase in MRI use over time, particularly in young adults [4], might be contributing to increased detection of stroke, rather than a true increase in incidence. This trend could be amplified by changes in the definitions of stroke and TIA, from a symptom-based diagnosis to an imaging-based one. In the past, a patient with transient or subtle neurological symptoms might have been diagnosed as having a transient ischemic attack (TIA), which would now be diagnosed as a stroke if diffusion-weighted abnormalities are noted on MRI [13]. This possibility is supported by evidence that increased MRI use leads to fewer missed stroke diagnoses [14, 15] and that this effect may be larger in younger adults [14, 16].

To explore the possibility that diagnostic classification or systems changes are contributing to trends in stroke in the young (18–44 years old), we sought to quantify the changes in patterns of neurologically focused ED visits, stroke/TIA diagnoses, and rates of MRI utilization from 1995 to 2015. We hypothesize that stroke/TIA diagnoses might be increasing in young adults due to increased use of MRI and that this effect would be specific to the young. Second, we hypothesize that amongst patients receiving a stroke or TIA diagnosis that stroke diagnoses would proportionately increase over time based on changes in the definition of TIA and trends in MRI utilization [17, 18].

## Methods

### Study design, setting, and participants

We performed a retrospective, serial, cross-sectional study on a nationally representative sample of all ED visits in the United States using National Hospital Ambulatory Medical Care Survey (NHAMCS) data from 1995 to 2000 and 2005–2015 to evaluate these two hypotheses.

NHAMCS is a set of annual, national probability sample data on utilization and provision of ambulatory care services in hospital emergency and outpatient departments and in ambulatory surgery centers. We utilized NHAMCS data, which are based on a complex survey design, from a national sample of visits to EDs in non-institutional general and short-stay hospitals, exclusive of Federal, military, and Veterans Administration hospitals, located in the 50 States and the District of Columbia. NHAMCS includes data such as demographics, visit characteristics such as patient’s reason for visit (RFV), procedural utilization, and provider’s diagnoses (see Additional File 1 for details on data collection and processing). This dataset was chosen based on its national representation and its inclusion of long-term time trends, RFV coding, and all ages of patients.

### Availability of data and materials

The datasets generated and/or analyzed during the current study are available through the CDC [19]. Because this study relies on publicly available data without personal identifiers, this study was deemed “not regulated” by the standards of the Institutional Review Boards of the University of Michigan Medical School and explicit review of the study protocol was waived (IRBMED, HUM00197055).

### Primary data analysis

#### Determining neurologic primary reason for ED visit (neuro RFV)

A strength of NHAMCS is inclusion of RFV which represents the patient’s complaint, symptom, or other reason for the visit. RFV data were coded in NHAMCS according to “A Reason for Visit Classification for Ambulatory Care” [20]. Our final Neuro RFV population was defined as visits by patients with a primary RFV of neurologically focused symptoms or concerns. From NHAMCS “A Reason for Visit Classification for Ambulatory Care” RFV code, we used the hierarchy of conditions listed under neurologically focused symptoms and concerns that we felt represented stroke/TIA diagnoses. We then edited this list via manual review of the top RFVs associated with the stroke/TIA population to identify RFVs that could plausibly represent stroke visits and to nearly all cases where a primary stroke diagnosis was ultimately assigned (see Additional Table 1).

**Table 1:** Study Population Baselines Characteristics

Demographics n (95% CI)	Neurological RFV ***n*** = 189 M (174 M - 204 M)	Stroke or TIA ***n*** = 9.55 M (8.68 M – 10.4 M)	Stroke ***n*** = 5.82 M (5.25 M - 6.39 M)	TIA ***n*** = 3.73 M (3.30 M - 4.17 M)	All Visits ***n*** = 2.01B (1.85B – 2.17B)
Age, mean yr (SD)	46 (23)	70 (15)	70 (15)	70 (15)	36 (24)
Female	59%	56%	55%	59%	54%
Race/ethnicity					
White	62%	73%	70%	78%	59%
Black	20%	13%	15%	9%	21%
Hispanic	11%	6%	6%	6%	13%
Other	7%	8%	9%	7%	7%
Insurance					
Private	30%	21%	20%	24%	32%
Medicare	26%	60%	60%	62%	17%
Medicaid	19%	7%	8%	6%	24%
Other	24%	11%	12%	9%	27%
MRI	2%	10%	10%	8%	< 1%
Age Distribution					
< 18	11%	< 1%	1%	< 1%	24%
18–44	40%	6%	5%	6%	41%
45–64	24%	26%	27%	25%	20%
65 +	25%	68%	67%	69%	15%
Comorbidities^a^					
Hypertension	32%	66%	65%	68%	22%
Diabetes	13%	27%	33%	19%	9%
CEBVD	7%	60%	60%	61%	3%
Hyperlipidemia	11%	37%	37%	37%	7%

*MRI* magnetic resonance imaging, *CEBVD* cerebrovascular disease, *SD* standard deviation

^a^Diabetes and CEBVD data were only available from 2009 and beyond. Hypertension and Hyperlipidemia data were only available from 2014 and beyond

#### Study populations

Our primary study population was the neurologically focused ED visit (Neuro RFV) population, defined as any patient visit to the ED with a neurologic symptom as the primary reason for visit. Our secondary study population was specifically the Stroke/TIA Population, defined as any patient visit to the ED that receives a primary diagnosis of stroke or TIA by the ED physician. We used International Classification of Diseases, Ninth Revision, Clinical Modification (ICD-9-CM) to determine visits by patients in whom the ED physician’s primary diagnosis was TIA (435.XX) or ischemic stroke (433.× 1, 434.× 1, 436.xx) [21–27]. Individual visits were included in either population based exclusively on their primary RFV or diagnosis. Of note, we are unable to determine if a specific stroke or TIA diagnosis was made on the basis of symptoms and/or imaging.

Our primary analysis was performed on these two different population subsets from 1995 to 2015, excluding the years 2001–2004 based on the unavailability of an individual MRI flag for these years (see Additional File 1). For our primary analysis we stratified population into four broader age groups (< 18 years, 18–44 years, 45–64 years, and > 65 years). “Young” was defined as 18–44 years old and “older adults” were defined as > 65 years old. ED diagnoses data were used to increase power and reduce bias through missing data. However, we also performed a sensitivity analysis using NHAMCS’s Hospital Discharge Diagnosis flag, which was available for 2005–2015, and identified visits where the primary discharge diagnosis after hospitalization was stroke or TIA using the ICD-9-CM codes described above. We compared agreement between ED diagnosis and hospital discharge diagnosis of stroke and TIA using Cohen’s Kappa statistic. We then repeated the analysis (described below for ED diagnoses) using the hospital discharge diagnoses of stroke and TIA.

We first summarized the characteristics of both study populations (Neuro RFV and Stroke/TIA) with percentages or means and standard deviations (SDs). We then explored the most common RFVs among the stroke/TIA population on the whole and then stratified across age categories. We also examined trends in the most common RFVs in the stroke/TIA population over time, examining top RFVs in three time periods (1995–2000, 2005–2009, and 2010–2015). Additionally, we examined the most common primary diagnoses that were assigned to the Neuro RFV population and how these varied by age group and time period. To further investigate migraine as a “stroke mimic”, we also investigated prevalence of all migraine diagnoses specifically in the Neuro RFV and stroke/TIA populations, stratified by sex and age group.

#### Trends in neuro RFVs

To understand whether the Neuro RFV population itself was changing over time, we also examined trends in Neuro RFV incidence by reporting survey estimates of the number of Neuro RFVs per 100,000 population per year and stratified by age category. Survey-weighted confidence intervals for incidence data were estimated with bootstrapping. For incidence calculations, population size estimates were obtained for each category of age groups using public US Census Bureau data. We also examined the changes in the proportion of Neuro RFVs out of all ED visits, stratifying by year and age category. We then built a logistic regression model to estimate how the proportion of Neuro RFVs in the total population changed over time using Neuro RFV as the dependent variable and time (year) as the independent variable. We repeated these analyses adjusting for race, sex, and insurance status (private vs. Medicare vs. Medicaid vs. other) to assess whether other factors that may influence Neuro RFVs and stroke/TIA diagnoses over time are contributing to differences in time trends. Finally, we repeated this analysis including an age category-time interaction term to assess whether time trends vary by age group.

We tested our first hypothesis, that stroke/TIA diagnoses are increasing amongst Neuro RFVs using a similar approach. Within the Neuro RFV population, we calculated the proportion that received a stroke or TIA diagnosis, assessed for adjusted and unadjusted time trends using survey-weighted logistic regression and estimated population incidence. To explore the relationship of these trends to MRI utilization, we examined how often MRIs were ordered/performed on the Neuro RFV population using a logistic model. This was examined over each year, stratified by age group, and adjusted for race, sex, and insurance status.

#### Trends in stroke/TIA diagnoses

To test our second hypothesis, we calculated the percentage of strokes and TIAs separately within the stroke/TIA population. To assess for adjusted time trends, we used a logistic model, examined diagnoses over each year, stratified by age group, and adjusted for race, sex, and insurance status. We also examined trends in incidence as explained above.

#### Guidelines for statistical analyses

All statistical analyses were conducted using Stata 14 (StataCorp, College Station, TX), as to consider the clustered nature of the sample, while following NHAMCS guidelines for survey data analysis (see Additional File 1) [28].

## Results

Over the 17-year study period (1995–2000; 2005–2015), 189 million ED visits (95% CI 174 M–204 M) with a neurologically focused primary RFV (Neuro RFV population) were identified (Table 1). Mean age was 46 ± 23 years, and 59% were female (95% CI 58–60%). 5.8 million ED visits with a primary stroke diagnosis (95% CI 5.3 M–6.4 M) were identified compared to 3.7 million primary TIA diagnosis (95% CI 3.3 M–4.2 M). Stroke and TIA patients were older, more likely to be insured by Medicare, and more likely to have comorbidities such as hypertension, diabetes, cerebrovascular disease, and hyperlipidemia. Compared to white patients, blacks had lower representation in the stroke/TIA population (13% in blacks) compared to the broader Neuro RFV population (20% blacks). The Neuro RFV population, compared to the total NHAMCS population, was older and had higher rates of comorbidities.

### Characterizing RFVs and diagnoses

Within the entire Neuro RFV population, the most common primary diagnoses were headache (12%), migraine (7%), dizziness and giddiness (5%), other convulsions (4%), and syncope and collapse (3%). There was little variation in these diagnoses over time. However, there were differences amongst age groups, with older age groups having lower proportions of headache diagnoses (see Additional Tables 2, 3 and 4). Within the stroke/TIA population, the most common primary RFVs were cerebrovascular disease (14%), (neurologic) weakness (11%), (anesthesia) loss of feeling (9%), general weakness (9%), and vertigo-dizziness (5%). There was little variation in these RFVs over time and between age groups (see Additional Tables 2, 3, and 4), with cerebrovascular disease being the top primary RFV regardless of time period or age group. However, for older age groups, cerebrovascular disease made up a lower proportion of the primary RFVs. Additionally, we found females in the Neuro RFV and stroke/TIA populations to more commonly have a diagnosis of migraine compared to males (see Additional File 1).

### Temporal trends in neuro RFVs

**Fig. 1:**
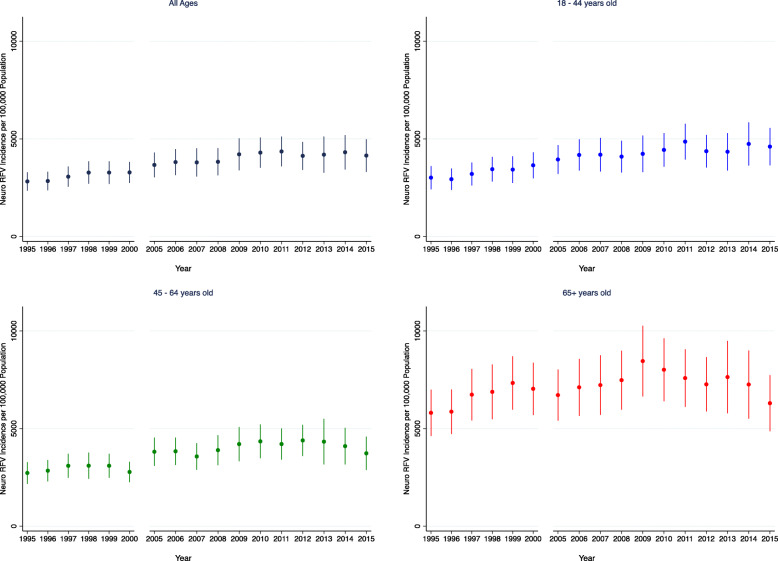
Neuro RFV Incidence by Age Group. The incidence of Neuro RFVs per 100,000 population by year and by age group (all ages, 18–44 years old, 45–64 years old, and 65+ years old). Error bars represent 95% confidence intervals. In the young (18–44 years, the incidence was + 111 Neuro RFVs/100,000 population/year (95% CI + 94 − + 130), whereas in older adults, incidence was + 70 Neuro RFVs/100,000 population/year (95% CI + 34 − + 108)

The Neuro RFV incidence (Fig. 1) was higher overall in absolute terms in older adults, with a significantly increasing trend over time in the young and older adults (young: + 111 Neuro RFVs/100,000 population/year, 95% CI + 94 − + 130; older adults: + 70 Neuro RFVs/100,000 population/year, 95% CI + 34 − + 108). Neuro RFV incidence rose faster in the young compared to older adults (*p* = 0.022) This finding was consistent in subgroup analyses (see Additional File 1 and Additional Figure 1).

### Evaluation of hypothesis 1: temporal trends of stroke/TIA diagnoses

**Fig. 2:**
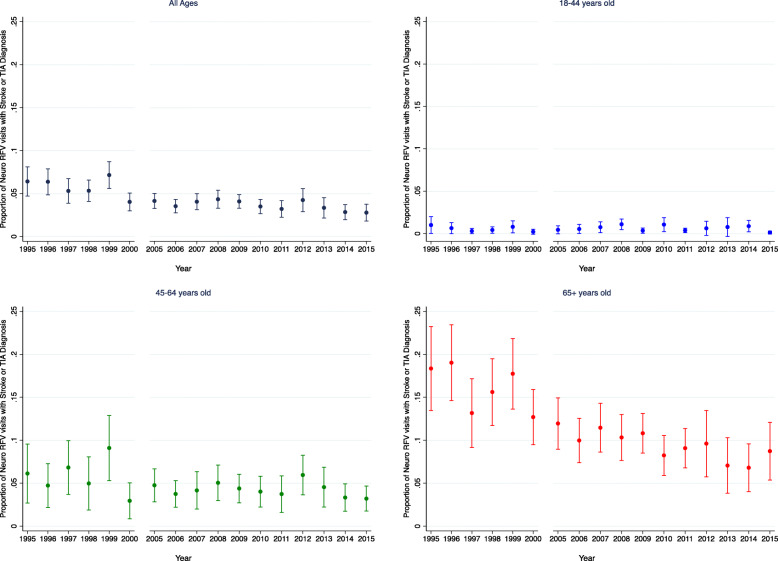
Stroke/TIA Diagnoses within Neuro RFV Population. The probability of a stroke/TIA diagnosis within the Neuro RFV population by year and by age group (all ages, 18–44 years old, 45–64 years old, and 65+ years old). Error bars represent 95% confidence intervals. For unadjusted analysis of all ages: OR = 0.962 (95% CI 0.951–0.973, *p* < 0.001), while for adjusted: OR = 0.960 (95% CI 0.948–0.971, p < 0.001). This downward trend was driven primarily by the older adult population (adjusted OR 0.952, 95% CI 0.939–0.965, p < 0.001) compared to the young (adjusted OR 0.997, 95% CI 0.954–1.042, *p* = 0.883)

The probability of a stroke/TIA diagnosis within the Neuro RFV population is shown in Fig. 2. There is a slight downward trend over time in both unadjusted (OR 0.962, 95% CI 0.951–0.973, *p* < 0.001) and adjusted (OR 0.960, 95% CI 0.948–0.971, p < 0.001) analyses. This downward trend was driven primarily by the older adult population (adjusted OR 0.952, 95% CI 0.939–0.965, p < 0.001) compared to the young (adjusted OR 0.997, 95% CI 0.954–1.042, *p* = 0.883).

### Evaluation of hypothesis 2: variation in the proportion of strokes and TIAs

**Fig. 3:**
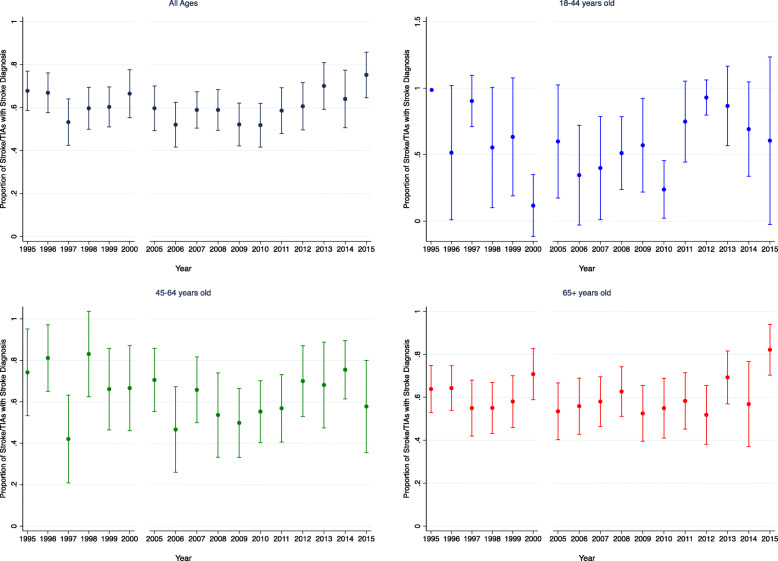
Proportion of Stroke/TIA Population with Stroke Diagnosis. The percent of stroke or TIA diagnoses with a primary stroke diagnosis by year and by age group (all ages, 18–44 years old, 45–64 years old, and 65+ years old). Error bars represent 95% confidence intervals. There is no evidence of a linear temporal trend in the all ages group (adjusted OR 1.001, 95% CI 0.982–1.021, *p* = 0.896) or trends in the young (adjusted OR 1.018, 95% CI 0.933–1.109, *p* = 0.692) or older adult populations (adjusted OR 1.005, 95% CI 0.982–1.029, *p* = 0.648). For the 18–44 years old group, the proportion of stroke/TIAs with stroke diagnosis was 1 in 1995, so this year was not included in the logistic model, and thus, lacks error bars

Within the stroke/TIA population, there is a general trend over time towards a higher proportion of strokes compared to TIAs, particularly after 2009 (Fig. 3). However, there was no clear evidence of a linear temporal trend (adjusted OR 1.001, 95% CI 0.982–1.021, *p* = 0.896) or trends over time in the young (adjusted OR 1.018, 95% CI 0.933–1.109, *p* = 0.692) or older adult populations (adjusted OR 1.005, 95% CI 0.982–1.029, *p* = 0.648). Stroke and TIA incidence were decreasing in older adults and stable in the young (see Additional File 1 and Additional Figure 2a-b).

### MRI utilization

MRI utilization rates for visits with a neurological primary RFV have increased throughout the 17-year period studied for all age groups (Additional Figure 3). Overall, MRI utilization increased over time (adjusted OR 1.078, 95% CI 1.057–1.099, *p* < 0.001). This temporal trend was more evident in older adults (adjusted OR 1.090, 95% CI 1.060–1.121, p < 0.001) compared to the young (adjusted OR 1.059, 95% CI 1.028–1.091, p < 0.001).

Trends were not substantially altered for any finding in sensitivity analyses using hospital discharge diagnoses of stroke and TIA, although effect sizes were attenuated, and confidence intervals widened. Comparing primary ED and hospital discharge diagnosis, we found a Cohen’s Kappa statistic of 0.58 (95% CI 0.56–0.60) indicating moderate agreement. (see Additional File 1).

### Population level estimates of false positive rates

Specifically, our data support two premises: (1) the prior probability of stroke is considerably higher amongst older adults than in young adults presenting with neurologic symptoms; and (2) the overall incidence of Neuro RFVs is increasing over time and increasing faster in the young compared to older adults. Using these premises, we can work out a hypothetical example. While it is very challenging to directly measure the number of false positives, by making some reasonable assumptions, we can estimate the size of this group. Based on those assumptions, it is credible that an increase in false positive strokes in the young may lead to a disproportionate overestimate of the incidence of stroke in the young **(**Table 2**)**.

**Table 2:** Estimating Population-Level Stroke/TIAs Diagnoses

Age Group	Year	Neuro RFVs (Thousands)	Stroke/TIA Prevalence (%)	True Positive Stroke/TIAs ^**a**^ (Thousands)	False Positive Stroke/TIAs ^**b**^ (Thousands)	Total Stroke/TIA Diagnoses (Thousands)
Young	1995	3300	1.0%	33	33	66
	2015	5300	0.6%	32	53	84
	Change			−3%	61%	27%
Older Adults	1995	2000	18%	360	16	376
	2015	3000	9%	270	27	297
	Change			−25%	69%	−21%

^a^True Positives = Number of Neuro RFVs x Stroke/TIA Prevalence

^b^False Positives = Number of Neuro RFVs x (1 - Specificity) x (1 - Stroke/TIA Prevalence)

Specificity of Stroke/TIA diagnosis = 99%

We estimate there were about 3.3 M Neuro RFVs in the young group and 2 M Neuro RFVs in older adults in 1995, and these numbers increased to 5.3 M and 3 M, respectively, in 2015. From our data, we can estimate that the stroke/TIA prevalence, or the prior probability of stroke/TIA for a visit with a Neuro RFV in the ED, decreased in both age groups, from 1% in 1995 to 0.6% in 2015 in younger adults and decreased from 18% in 1995 to 9% in 2015 in older adults. Based on this declining prevalence over time, we can roughly estimate the following: young adults had 33,000 true positive strokes/TIAs (Number of Neuro RFVs × Prevalence), 33,000 false positive stroke/TIAs (Number of Neuro RFVs × (1-Specificity) × (1-Prevalence)), and 66,000 total stroke/TIA diagnoses in 1995, while the older adult group would have had 360,000 true positive stroke/TIAs, 16,000 false positive stroke/TIAs, and 376,000 total stroke/TIA diagnoses. Then in 2015 in the young group, it can be estimated that there were 32,000 true positive stroke/TIAs (3% relative decrease), 53,000 false positive stroke/TIAs (61% relative increase), and 84,000 total stroke/TIA diagnoses (27% apparent increase). In 2015, the elderly group were estimated to have 270,000 true positive stroke/TIAs (25% relative decrease), 27,000 false positive stroke/TIAs (69% increase), and 297,000 total stroke/TIA diagnoses (21% relative decrease). In other words, while the apparent rate of total stroke/TIAs may be rising in the young, it may be the rising number of false positive strokes as a result of higher total ED evaluations for Neuro RFVs that are driving this apparent increase. This hypothetical assumes a specificity of 99% for correctly diagnosing stroke/TIA in an ED visit in both age groups. This may underestimate the relative contribution of false positive strokes/TIAs because the specificity of an ED evaluation for this diagnosis is likely less than 99%.

## Discussion

In a nationally representative sample of ED visits over 17 years, we did not find evidence to support our primary hypotheses. There was neither a differential increase in the proportion of young people with strokes amongst those presenting with neurologic complaints compared to older adults nor evidence of differential classification of TIA to stroke over time in any age group. We also did not find a disproportionate rise in MRI use for Neuro RFVs in older adults compared to young adults. Our analysis did identify, however, a robust trend that may play an important role in the apparent increase in stroke in the young. Specifically, given the lower pre-test probability of a stroke in younger adults and the rise in Neuro RFVs, an increase in false positive strokes in the young may lead to a disproportionate overestimate of the incidence of stroke in this population.

To be clear, these data do not directly measure trends in either true or false positive strokes, rather they measure population-level parameters that enable inferences about the rate of true and false positive strokes/TIAs. Importantly, this logic would apply even if stroke were assessed via any algorithm with less than 100% specificity (even a gold-standard algorithm) as opposed to a claims-based definition as was applied here. Moreover, as our hypothetical example illustrates, under reasonable assumptions it is plausible that the magnitude of the increase in false positive strokes would be large enough to lead a mistaken impression of increasing stroke in the young even if the number of strokes is steady or declining. The potential significance of changing rates of false positive stroke is complex. While increasing false positive stroke rates may lead to misestimation of epidemiologic trends, they may also reflect other positive trends. For example, public awareness and education campaigns have sought to encourage patients to emergently present to the ED with acute neurologic symptoms. To the extent these campaigns increase neurologic presentations, it is quite likely that false positives will almost inevitably increase as well. As such, increasing false positives may represent a rational societal trade off — a reasonable price to pay to increase thrombolysis rates.

A possible alternate explanation for increasing incidence of stroke in the young is that the increase may be a true increase due to changes either in risk factor profile or etiology of stroke in this population. While risk factor changes may be due to changes in measurement over time [12], the best way to investigate the theory that other subtypes of stroke may be increasing would be to examine high quality population data systematically characterizing ED presentations with gold-standard stroke diagnostic algorithms and stroke subtyping. However, true changes in incidence in stroke in the young would not explain the large rise in Neuro RFVs seen in this population.

These data also suggest the possibility that race differences amongst young adults with stroke may be misestimated. Large race differences in stroke incidence have been consistently observed and are particularly prominent in young adults [29]. We found that blacks had lower representation in the stroke/TIA population (13%) compared to the broader Neuro RFV population (20%), which suggests that blacks, upon coming to the ED with a primary neurologic complaint, are less likely to receive a stroke/TIA diagnosis compared to whites. If diagnostic accuracy does not differ by race, then the higher number of ED visits amongst young Blacks would imply more false positive diagnoses amongst Blacks and thus a possible overstatement of race-differences in incidence. Conversely, these data suggest the possibility that diagnostic accuracy may differ by race. If young Blacks have the same (or higher) prior probability of a stroke diagnosis at the time of ED presentation than whites, then these data are consistent with underdiagnosis of stroke amongst young Blacks and thus underestimation of racial differences in incidence. Improving our understanding of racial differences in the diagnostic process, particularly amongst young adults, is essential to interpreting these findings.

There are several limitations to this study. First, the reason for visit codes applied to identify neurologic symptom presentations have not been previously validated. While it is reassuring that the same coding schema and approach to collecting these data were applied over the entire study period, we cannot exclude the possibility that different reasons for visit may be assigned to similar presentations in 2015 and 1995. Second, for this study, we were also unable to determine if a specific stroke or TIA diagnosis was made on the basis of symptoms and/or imaging. The MRI flag used only indicated if an MRI was ordered and/or obtained in the ED, but not whether it was obtained prior to a diagnosis being made. While administrative definitions of stroke have reasonable accuracy [21, 22, 30, 31] and have maintained reasonable accuracy over time, it is possible that they have subtly improved over time, perhaps due to the dissemination of MRI. However, prior work suggests this is unlikely to be a large effect [32]. Given the increase in MRI rates over time, and high sensitivity of MRI to prior or non-ischemic lesions, it is also possible that the real-world effect is to increase false positive diagnoses and future work to explore how MRI findings and diagnoses vary in a large population of ED presentations may be informative. TIA ED diagnoses can also have poor interrater reliability. This analysis also does not explicitly account for changes in formal definitions of stroke and TIA from before and after 2009. However, by combing stroke and TIAs into one category, we are able to indirectly account for this change as the change in definition would lead to reclassification from one to the other. Third, our primary data comes solely from the primary ED diagnosis, which does not take into account strokes that may be diagnosed secondarily outside of the ED. When analyzing hospital discharge diagnoses, which differ from ED diagnoses maybe due to incomplete NHAMCS records, more intense evaluation in the inpatient setting, or due to incomplete ED records given the recent trend to directly admit stroke patients to inpatient services, our trends described above were all attenuated. Additionally, while we do not adjust trends for known cerebrovascular comorbidities, prior work suggests that there has not been a true change in risk factors in the young but rather a change in risk factor diagnoses [12].

We anticipate several next steps to better clarify our findings. Given the trends we described above were attenuated when using hospital discharge diagnosis data, exploring how trends are affected with gold-standard stroke diagnoses may be informative. Additionally, further research is needed to determine the effect of current stroke prevention campaigns targeted towards the young and where and how to target our efforts moving forward.

## Conclusions

We found, but did not anticipate, increased incidence of neurologically focused ED visits in young and older adults. Given the lower pre-test probability of a stroke in younger adults, our data suggest that false positive stroke diagnoses may be increasing and may be increasing more rapidly in the young than in older adults. Thus, increasing false positive diagnoses in the young might be a contributing factor to the observed increases in stroke incidence in this population. If stroke in the young is truly rising, then this represents a failure of our healthcare system to understand and serve the needs of a large segment of our population. However, we present an alternate theory that merits further exploration.

## Supplementary Information

**Additional file 1.**

## Data Availability

The datasets generated and/or analyzed during the current study are available through the CDC, [https://www.cdc.gov/nchs/ahcd.htm].

## Abbreviations

ED: Emergency department
CI: Confidence interval
OR: Odds ratio
RFV: Reason for visit
Neuro RFV: Neurologically focused reason for visit
MRI: Magnetic resonance imaging
TIA: Transient ischemic attack
NHAMCS: National Hospital Ambulatory Medical Care Survey
CDC: Center for Disease Control
ICD-9-CM: International Classification of Diseases, Ninth Revision, Clinical Modification
