# Supplementary material for: Increasing false positive diagnoses may lead to overestimation of stroke incidence, particularly in the young: a cross-sectional study
Source: BMC Neurol. 2021 Apr 8;21:152. doi: 10.1186/s12883-021-02172-1 (PMC8028807; doi:10.1186/s12883-021-02172-1)
Supplement: Supplementary file 1 — Additional file 1. [file 12883_2021_2172_MOESM1_ESM.docx]

**ADDITIONAL INFORMATION**

**Increasing false positive diagnoses may lead to overestimation of stroke incidence, particularly in the young: a cross-sectional study**

**Short Title:** Investigating stroke incidence in the young

Abhinav J. Appukutty, BS^1^, Lesli E. Skolarus, MD^2,3^, Mellanie V. Springer, MD^2^, William J. Meurer, MD^2,4^ and James F. Burke, MD^2,5,*^

^1^University of Michigan Medical School, 1500 E Medical Center Dr, Ann Arbor, MI 48109 USA

^2^Stroke Program, University of Michigan Medical School, 1500 E Medical Center Dr, Ann Arbor, MI 48109 USA

^3^School of Public Health, University of Michigan, 1415 Washington Heights, Ann Arbor, MI 48109 USA

^4^Emergency Department, University of Michigan Medical School, 1500 E Medical Center Dr, Ann Arbor, MI 48109 USA

^5^Department of Neurology, VA Ann Arbor Healthcare System, Ann Arbor, MI 48105 USA

**^*^Corresponding Author:**

Academic title: Associate Professor of Neurology, University of Michigan Medical School

E-mail: jamesbur@med.umich.edu

Phone: 734-764-5871

Mailing: IHPI Clinician Scholars Program, 2800 Plymouth Rd, Bldg 14, Rm G105, Ann Arbor, MI 48109

**This document includes:**

1. Additional Methods
2. Additional Results
3. Additional Tables 1 – 4
4. Additional Figures 1 – 3 and Figure Legends

I. Additional Methods

***Data Collection and Processing***

Hospital staff or Census Bureau field representatives complete a patient record form for each sampled visit based on medical record information. Sample hospitals are randomly assigned to 16 panels that rotate across 4-week reporting periods, with each hospital surveyed approximately once every 15 months. On average, approximately 88% of sampled hospitals participated in the survey, and about 88% of sampled EDs provided complete information on their sample visits, for a total unweighted response rate of 75%. Detailed descriptions of data collection, abstraction, and analysis procedures is available for review through the CDC.

***Other Measures***

NHAMCS directly abstracts whether an MRI was ordered and/or provided. However, for the years of 2000-2004, receipt of MRI and CT were not differentiated and thus, data were excluded for those years. We characterized patient comorbidities using NHAMCS’s directly abstracted comorbidity variables. Due to inconsistencies in which comorbidities are captured over time, diabetes and cerebrovascular disease (as a comorbidity) were only tracked from 2009 onwards and hypertension and hyperlipidemia were only tracked from 2014 onwards

***Guidelines for Statistical Analysis***

The survey data were analyzed using sampled visit weights that have been adjusted by the National Center of Health Statistics (NCHS) for survey nonresponse within time of year, geographic region, and urban/rural and ownership designations, which results in an unbiased national estimate of ED visit data.

II. Additional Results

*Characterizing RFVs and Diagnoses –* We investigated prevalence of migraine diagnosis specifically in Neuro RFVs and the stroke/TIA population, stratified by sex and age group. Of Neuro RFVs, 8.8% (95% CI 8.2% - 9.4%) had any migraine diagnosis, and of these, 80.9% (95% CI 79.1% - 82.5%) were female. In the young, 14.8% (95% CI 13.7% - 15.9%) of Neuro RFVs had any migraine diagnosis, and of these, 80.8% (95% CI 78.6% - 82.9%) were female. In older adults, 0.9% (95% CI 0.6% - 1.2%) of Neuro RFVs had any migraine diagnosis, and of these, 77.1% (95% CI 56.9% - 89.6%) were female. Of the stroke/TIA diagnosis group, 0.1% (95% CI 0.0 - 0.4%) had a secondary or tertiary diagnosis of migraine, and of these, 90.5% (95% CI 48.0% - 99.0%) were female. In the young, 0.1% (95% CI 0.0 - 0.4%) of the stroke/TIA population had a secondary or tertiary migraine diagnosis, and of these 100% were female. There were no older adults with a stroke/TIA diagnosis that received a secondary or tertiary migraine diagnosis.

*Temporal Trends in Neuro RFVs –* To explore whether there may be variation in RFV temporal trends within demographic subgroups, we also analyzed percent of all ED visits with a Neuro RFV by year and by age group. We found Neuro RFVs increased over time across all age groups We then analyzed the percent of all ED visits with a Neuro RFV by year and by age group (Supplementary Fig. 1**)**. Across all age groups, Neuro RFVs increased over time (OR 1.010 per year, 95% CI 1.007 – 1.013, p < 0.001). This trend persisted after adjusting for the sex, race, and insurance status (OR 1.008 per year, 95% CI 1.005 – 1.011, p < 0.001). This trend also differed by age with a more rapid increase in Neuro RFVs in the in the young (OR 1.007 per year, 95% CI 1.003 – 1.011, p = 0.001) compared to the older adults (OR 1.001, 95% CI 0.998 – 1.005, p = 0.504), although this was attenuated after adjusting for sex, race, and insurance status (OR 1.003 per year, 95% CI 0.999 – 1.007, p = 0.133 in young; OR 1.000, 95% CI 0.996 – 1.004, , p = 0.876 in older adults).

*Evaluation of Hypothesis 2: Estimated Incidence of Stroke and TIA* – The estimated incidence of stroke diagnoses (Supplementary Figure 2a) appeared to be relatively steady over time for the young compared to a decreasing trend over time in older adults (young: +0 stroke diagnoses/100,000 population/year, 95% CI -1 – +2; older adults: -29 stroke diagnoses/100,000 population/year, 95% CI -40 – -18), with similar relationships found for the estimated rate of TIA diagnoses shown in Supplementary Figure 2b (young: +0 TIA diagnoses/100,000 population/year, 95% CI +0 – +1; older adults: -21 TIA diagnoses/100,000 population/year, 95% CI -30 – -12).

*Sensitivity Analysis using Hospital Discharge Diagnoses Data* – Using 2005-2015 data, all trends involving stroke/TIA diagnoses were generally similar when using primary hospital discharge diagnoses rather than ED diagnoses with no major shifts in the direction of the trends, although all effect sizes were attenuated and confidence intervals widened. Of note, there was no admission in 74% of cases and no documented hospital discharge diagnosis for 16% of cases.

This trend was true for fraction of stroke/TIAs within the Neuro RFV population (young: adjusted OR 0.968, 95% CI: 0.863 – 1.086, p = 0.585; older adults: adjusted OR 0.979, 95% CI: 0.933 – 1.027, p = 0.383), the proportion of strokes within the stroke/TIA population (young: adjusted OR 1.337, 95% CI: 0.887 – 2.016, p = 0.165; older adults: adjusted OR 0.972, 95% CI: 0.895 – 1.056, p = 0.505), incidence of stroke (young: +1 stroke diagnoses/100,000 population/year, 95% CI: -1 – +2; older adults: -14 stroke diagnoses/100,000 population/year, 95% CI: -28 – +1), and incidence of TIA (young: -1 TIA diagnoses/100,000 population/year, 95% CI: -2 – +0; older adults: -1 TIA diagnoses/100,000 population/year, 95% CI: -14 – +14).

**II. Additional Tables**

| **Additional Table 1. Defining Neuro RFV Population** | | | | |
| --- | --- | --- | --- | --- |
| **RFV1 Code** | **Reason for Visit** | **Original Neuro RFV Definition** | **Top 25 RFVs from Stroke/TIA Population** | **RFVpop: Final Adjusted Definition** |
|  |  |  |  |  |
|  |  |  |  |  |
|  |  |  |  |  |
| 2525.0 | Cerebrovascular Disease |  | X | ✓ |
| 1230.0 | Weakness (neurologic) | X | X | ✓ |
| 1220.1 | Loss of feeling (anesthesia) | X | X | ✓ |
| 1020.0 | General weakness | X | X | ✓ |
| 1225.0 | Vertigo - dizziness | X | X | ✓ |
| 1235.2 | Slurring | X | X | ✓ |
| 1165.0 | Other problems related to psycho… |  | X | ✓ |
| 1240.0 | Other symptoms referable to the nervo.. | X | X | ✓ |
| 1235.0 | Disorders of speech/speech disturbance | X | X | ✓ |
| 1210.0 | Headache, pain in head | X | X | ✓ |
| 5840.0 | Unconscious on arrival |  | X | ✓ |
| 5810.0 | Accident, NOS |  | X |  |
| 1220.3 | Abnormal sensation (paresthesia) | X | X | ✓ |
| 1050.1 | Chest pain, soreness |  | X |  |
| 1095.0 | Disorders of motor function |  | X | ✓ |
| 1415.0 | Shortness of Breath |  | X |  |
| 2370.0 | Other and unspec diseases of the nervous sys |  | X | ✓ |
| 1945.4 | Weakness of arm |  | X | ✓ |
| 1305.2 | Diminished vision | X | X | ✓ |
| 5841.0 | State of consciousness not specified |  | X | ✓ |
| 1030.0 | Fainting (syncope) |  | X |  |
| 2510.0 | Hypertension |  | X |  |
| 5842.0 | Altered level of consciousness |  | X | ✓ |
| 1205.0 | Convulsions | X | X | ✓ |
| 1920.4 | Weakness of leg |  | X | ✓ |
| 1200-1259 | Symptoms referable to the nervous system | X |  | ✓ |
| 1020.0 | General weakness | X |  | ✓ |
| 1305.1-.4 | Visual dysfunctions | X |  | ✓ |
| 1340.4 | Abnormal eyelid movements | X |  | ✓ |
| 2365.0 | Migraine headaches | X |  | ✓ |
| 3345.0 | Diagnostic radiological abnormalities | X |  |  |
| 6400.0 | Radiological abnormalities | X |  |  |
| 6700.0 | Abnormal test results | X |  |  |
| Green highlight = RFV included in final definition of Neuro RFV population | | | | |

| **Additional Table 2. Most Common RFVs and Primary Diagnoses Overall** | | |
| --- | --- | --- |
| **Rank** | **Top Primary RFVs within stroke/TIA pop (%)** | **Top Primary Diagnoses within Neuro RFV pop (%)** |
| 1 | Cerebrovascular disease (14%) | Headache (12%) |
| 2 | (Neurologic) weakness (11%) | Migraine, unspec. (7%) |
| 3 | (Anesthesia) Loss of feeling (9%) | Dizziness & giddiness (5%) |
| 4 | General weakness (9%) | Other convulsions (4%) |
| 5 | Vertigo - dizziness (5%) | Syncope and collapse (3%) |
| 6 | Slurring (5%) | Other malaise and fatigue (2%) |
| 7 | Other symptoms/probs related to psycho… (4%) | Unspecified essential hypertension (2%) |
| 8 | Other symptoms referable to the nervous sys (4%) | Unspecified transient cerebral ischemia (2%) |
| 9 | Disorders of speech, speech disturbance (4%) | Altered mental status (2%) |
| 10 | Headache, pain in head (3%) | Cerebral art. occlusion, unspec. w/ infarction (1%) |
| 11 | Unconscious on arrival (3%) | Head injury, unspecified (1%) |
| 12 | Accident, NOS (2%) | Urinary tract infection, site not specified (1%) |
| 13 | (Paresthesia) Abnormal sensation (2%) | Depressive disorder, not elsewhere classified (1%) |
| 14 | Chest pain (1%) | Disturbance of skin sensation (1%) |
| 15 | Disorders of motor functions (1%) | Epilepsy, unspecified (1%) |
| 16 | Shortness of breath (1%) | Alcohol abuse, unspecified (1%) |
| 17 | Other and unspec diseases of the nervous sys (1%) | Contusion of face, scalp, and neck, exc. eyes (1%) |
| 18 | Weakness of arm (1%) | Ischemic stroke (Acute, but ill-defined CVD) (1%) |
| 19 | Diminished vision (1%) | Unspecified sinusitis (chronic) (1%) |
| 20 | State of consciousness not specified (1%) | Unspecified viral infection (1%) |
| 21 | (Syncope) Fainting (1%) | Pneumonia, organism unspecified (1%) |
| 22 | Hypertension (1%) | Suicidal ideation (1%) |
| 23 | Altered level of consciousness (1%) | Unspecified transport accident (1%) |
| 24 | Convulsions (1%) | Convulsions (1%) |
| 25 | Weakness of leg (1%) | Transfusion associated circulatory overload (1%) |

| **Additional Table 3. Most Common RFVs and Primary Diagnoses by Time Period** | | | |
| --- | --- | --- | --- |
| **Time Period** | **Rank** | **Top Primary RFVs within stroke/TIA pop (%)** | **Top Primary Diagnoses within Neuro RFV pop (%)** |
| 1995 - 2000 | 1 | Cerebrovascular disease (10%) | Headache (11%) |
|  | 2 | (Anesthesia) Loss of feeling (10%) | Migraine, unspec. (9%) |
|  | 3 | (Neurologic) weakness (10%) | Dizziness & giddiness (4%) |
|  | 4 | General weakness (7%) | Ischemic stroke (Acute, ill-defined CVD) (3%) |
|  | 5 | Vertigo - dizziness (7%) | Other convulsions (3%) |
| 2005 - 2009 | 1 | Cerebrovascular disease (15%) | Headache (12%) |
|  | 2 | (Neurologic) weakness (12%) | Migraine, unspec. (8%) |
|  | 3 | General weakness (9%) | Dizziness & giddiness (5%) |
|  | 4 | (Anesthesia) Loss of feeling (9%) | Other convulsions (5%) |
|  | 5 | Vertigo - dizziness (5%) | Syncope and collapse (3%) |
| 2010 - 2015 | 1 | Cerebrovascular disease (17%) | Headache (13%) |
|  | 2 | (Neurologic) weakness (12%) | Dizziness & giddiness (6%) |
|  | 3 | General weakness (10%) | Migraine, unspec. (6%) |
|  | 4 | (Anesthesia) Loss of feeling (8%) | Other convulsions (4%) |
|  | 5 | Slurring (5%) | Other malaise and fatigue (3%) |

| **Additional Table 4. Most Common RFVs and Primary Diagnoses by Age Group** | | | |
| --- | --- | --- | --- |
| **Age Group** | **Rank** | **Top Primary RFVs within stroke/TIA pop (%)** | **Top Primary Diagnoses within Neuro RFV pop (%)** |
| < 18 | 1 | Cerebrovascular disease (27%) | Headache (12%) |
|  | 2 | Other symptoms referrable to nervous sys (16%) | Other convulsions (8%) |
|  | 3 | Migraine headache (10%) | Head injury, unspecified (5%) |
|  | 4 | Stiffness, site unspecified (7%) | Migraine, unspec. (3%) |
|  | 5 | Other and unspec diseases of the nervous sys (7%) | Unspecified viral infection (3%) |
| 18 - 44 | 1 | Cerebrovascular disease (19%) | Headache (18%) |
|  | 2 | (Neurologic) weakness (14%) | Migraine, unspec. (13%) |
|  | 3 | (Anesthesia) Loss of feeling (11%) | Other convulsions (5%) |
|  | 4 | Vertigo - dizziness (6%) | Dizziness & giddiness (4%) |
|  | 5 | Headache, pain in head (5%) | Depressive disorder, not elsewhere classified (2%) |
| 45 - 64 | 1 | Cerebrovascular disease (15%) | Headache (11%) |
|  | 2 | (Anesthesia) Loss of feeling (14%) | Migraine, unspec. (8%) |
|  | 3 | (Neurologic) weakness (10%) | Dizziness & giddiness (6%) |
|  | 4 | General weakness (8%) | Other convulsions (4%) |
|  | 4 | Headache, pain in head (7%) | Unspecified essential hypertension (3%) |
| 65+ | 1 | Cerebrovascular disease (13%) | Dizziness & giddiness (7%) |
|  | 2 | (Neurologic) weakness (11%) | Other malaise and fatigue (5%) |
|  | 3 | General weakness (10%) | Transient cerebral ischemic attack (4%) |
|  | 4 | (Anesthesia) Loss of feeling (7%) | Syncope and collapse (4%) |
|  | 5 | Vertigo - dizziness (5%) | Headache (4%) |

**III. Additional Figures**

**Additional Figure 1: Proportion of Primary RFVs with Neurologic Focus by Age Group**

The percent of all ED visits with a Neuro RFV by year and by age group (all ages, 18-44 years old, 45-64 years old, and 65+ years old). Error bars represent 95% confidence intervals. Across all age groups, Neuro RFVs increased over time (OR 1.010 per year, 95% CI 1.007 – 1.013, p < 0.001). This trend attenuated slightly, but persisted after adjusting for the sex, race, and insurance status (OR 1.008 per year, 95% CI 1.005 – 1.011, p < 0.001). This trend also differed by age with a more rapid increase in Neuro RFVs in the in the young (OR 1.007 per year, 95% CI 1.003 – 1.011, p = 0.001) compared to the older adults (OR 1.001, 95% CI 0.998 – 1.005, p = 0.504), although both trends attenuated after adjusting for sex, race, and insurance status (OR 1.003 per year, 95% CI 0.999 – 1.007, p = 0.133 in young; OR 1.000, 95% CI 0.996 – 1.004, , p = 0.876 in older adults).

**** ****

a

b

**Additional Figure 2a-b: Stroke and TIA Incidence by Age Group**

The incidence of stroke (top) and TIA (bottom) per 100,000 population by year and by age group (all ages, 18-44 years old, 45-64 years old, and 65+ years old). Error bars represent 95% confidence intervals. The estimated incidence of stroke diagnoses (top) appeared to be relatively steady over time for the young compared to a decreasing trend in older adults (young: +0 stroke diagnoses/100,000 population/year, 95% CI -1 – +2; older adults: -29 stroke diagnoses/100,000 population/year, 95% CI -40 – -18), with similar relationships found for the estimated rate of TIA diagnoses (bottom), with the young having +0 TIA diagnoses/100,000 population/year (95% CI +0 – +1) and older adults having -21 TIA diagnoses/100,000 population/year (95% CI -30 – -12).

**Additional Figure 3: Utilization of MRI for Neurological RFVs by Age Group**

MRI utilization rates for visits with a neurological primary RFV by year and by age group (all ages, 18-44 years old, 45-64 years old, and 65+ years old). Error bars represent 95% confidence intervals. Overall, MRI utilization increased over time (adjusted OR 1.078, 95% CI 1.057 – 1.099, p < 0.001). This trend was more evident in older adults (adjusted OR 1.090, 95% CI 1.060 – 1.121, p < 0.001) compared to the young (adjusted OR 1.059, 95% CI 1.028 – 1.091, p < 0.001).
